# Increased paired box transcription factor 8 has a survival function in Glioma

**DOI:** 10.1186/1471-2407-14-159

**Published:** 2014-03-06

**Authors:** Noelyn Hung, Yu-Jen Chen, Ahmad Taha, Magnus Olivecrona, Ronald Boet, Anna Wiles, Tracy Warr, Alisha Shaw, Ramona Eiholzer, Bruce C Baguley, Michael R Eccles, Antony W Braithwaite, Martin MacFarlane, Janice A Royds, Tania Slatter

**Affiliations:** 1 Department of Pathology, University of Otago, Dunedin, New Zealand; 2 Dunedin Public Hospital, Dunedin, New Zealand; 3 Department of Pharmacology and Clinical Neuroscience, Neurosurgery, Umea University, Umea, Sweden; 4 Christchurch Hospital, Christchurch, New Zealand; 5 Department of Medicine, Dunedin School of Medicine, University of Otago, Dunedin, New Zealand; 6 Department of Molecular Neuroscience, Institute of Neurology, National Hospital for Neurology and Neurosurgery, London, UK; 7 Auckland Cancer Society Research Centre, Faculty of Medical and Health Sciences, University of Auckland, Auckland, New Zealand; 8 Children’s medical research Institute, University of Sydney, Westmead, Australia

**Keywords:** PAX8, Glioblastoma, Glioma, Telomere maintenance mechanism, Telomerase, ALT, BCL2, Cell survival

## Abstract

**Background:**

The molecular basis to overcome therapeutic resistance to treat glioblastoma remains unclear. The anti-apoptotic b cell lymphoma 2 (*BCL2*) gene is associated with treatment resistance, and is transactivated by the paired box transcription factor 8 (PAX8). In earlier studies, we demonstrated that increased *PAX8* expression in glioma cell lines was associated with the expression of telomerase. In this current study, we more extensively explored a role for *PAX8* in gliomagenesis.

**Methods:**

PAX8 expression was measured in 156 gliomas including telomerase-negative tumours, those with the alternative lengthening of telomeres (ALT) mechanism or with a non-defined telomere maintenance mechanism (NDTMM), using immunohistochemistry and quantitative PCR. We also tested the affect of PAX8 knockdown using siRNA in cell lines on cell survival and BCL2 expression.

**Results:**

Seventy-two percent of glioblastomas were PAX8-positive (80% telomerase, 73% NDTMM, and 44% ALT). The majority of the low-grade gliomas and normal brain cells were PAX8-negative. The suppression of *PAX8* was associated with a reduction in both cell growth and *BCL2*, suggesting that a reduction in *PAX8* expression would sensitise tumours to cell death.

**Conclusions:**

PAX8 is increased in the majority of glioblastomas and promoted cell survival. Because PAX8 is absent in normal brain tissue, it may be a promising therapeutic target pathway for treating aggressive gliomas.

## Background

Glioblastomas are the most common histological subtype among all the malignant brain tumours [1,2]. With the distinct molecular subtypes of glioblastoma recently characterised, the hope of new glioblastoma therapeutics is imminent [3-5]. Paired box-containing (PAX) transcription factors are largely expressed during development and at low levels in adult tissue [6]. Aberrant *PAX* gene expression is present in multiple cancer types, including cancers of the lymphoid tissue, thyroid, kidney, breast, and endometrium [7-10]. Paired box-containing proteins also possess many tumour-promoting functions, such as the promotion of cell survival and anti-apoptotic properties, because a reduction in *PAX* gene expression induces apoptosis in normal and tumour cells [11-15].

*PAX8* is expressed at the midbrain-hindbrain junction during brain development and is virtually absent in the adult brain [16]. In earlier studies involving PAX8 and glioblastomas, we found increased *PAX8* expression in tumours using a small panel of 14 telomerase-positive tumours and cell lines [14,17]. The tumour-promoting functions of *PAX8* include the ability to transform cells and to form tumours in mice [18], an increased telomerase activity [17], and the promotion of cell cycle progression [19]. The genes upregulated by PAX8 include *b cell lymphoma 2 (BCL2)* and *Wilms tumour 1 (WT1)*. High-grade gliomas have a higher *WT1* expression level compared with low-grade gliomas [20], and *BCL2* is associated with the higher tumour grades, poorer patient survival, and the conferring of treatment resistance through its own action and the action of other gene family members [21-25].

The prevalence of increased *PAX8* expression has not been extensively explored in glioma, especially with regard to the effect of increased *PAX8* expression in telomerase-negative gliomas. Here, we surveyed the *PAX8* expression in a range of brain tumours, including different grades of gliomas and varieties of telomere maintenance mechanisms.

## Methods

### Tumour samples

Brain tumours were procured during surgery from patients admitted to New Zealand hospitals. The Multi-region Ethics Committee, New Zealand, approved this study, and all patients provided written informed consent. Each hospital made the original histological diagnoses, which were subsequently reviewed by consultant neuropathologists at the referral centres, and confirmed by the study consultant neuropathologist who was blind to the original diagnoses. The glioblastomas used in this already had the telomere maintenance mechanism established as part of previous studies or had the telomere maintenance mechanism typed in the current study by methods outlined elsewhere [26,27]. Briefly, ALT positive tumours had heterogeneous telomere lengths by terminal restriction fragment (TRF) analysis and were positive for ALT associated promyelocytic leukaemia nuclear bodies (APB), but were negative for telomerase activity using the telomere repeat amplification protocol (TRAP) assay. Telomerase positive tumours were positive for telomerase activity using the TRAP assay and did not have APBs or heterogeneous telomere lengths by TRF analysis. NDTMM tumours did not have heterogeneous telomeres by TRF analysis and were negative for telomerase activity by TRAP analysis.

### Immunohistochemistry (IHC)

Paraffin-embedded brain tissues were mounted on microscope slides and were subjected to heat-mediated antigen retrieval. Primary antibodies raised against PAX8 (MRQ-50 and PAX8 [polyclonal] antibodies, Cell Marque, Rocklin, CA) and PAX5 (clone 24, Cell Marque, Rocklin, CA) were used and detected using the EDL (Dako, Glostrup, Denmark) and DAB methods. PAX5- or PAX8-positive cells were detected with light microscopy, and the percentage of positive cells per 1000 tumours cells was calculated (DM 2000 microscope, DFC 295 camera and Application Suite software, version 3.5.0, Leica, Solms, Germany). The slides were assessed by three authors (AS, NH and TS) independently. A tumour was considered positive for PAX8 or PAX5 when 10% or more of the tumour nuclei were moderately or faintly stained by IHC.

### Quantitative PCR

Total RNA was extracted from glioma specimens using the RNeasy Lipid Tissue Mini Kit (Qiagen, GmbH, Germany) following the manufacturer’s instructions. For quantitative PCR (QPCR), the first-strand cDNA from 50 ng RNA was used. Relative quantification of the *PAX8* transcripts and the two housekeeping genes, *glyceraldehyde 3-phosphate dehydrogenase (GAPDH)* and *hypoxanthine phosphoribosyltransferase 1 (HPRT1)* by real time PCR was determined utilising the SYBR-green detection protocol and the ABI PRISM 7000 or 7300 Sequence Detection System (Life Technologies, Carlsbad, CA). The primer sequences used were as follows:

*PAX8* forward primer: 5′-TTTGCTTGGCTCTTTCTACACCTC-3′

*PAX8* reverse primer: 5′-GAATGTCTGTTTTAAGCTCCCTGG-3′

*GAPDH* forward primer: 5′-TGCACCACCAACTGCTTAGC-3′

*GAPDH* reverse primer: 5′-GGCATGGACTGTGGTCATGAG-3′

*HPRT1* forward primer: 5′-TGACACTGGCAAAACAATGCA-3′

*HPRT1* reverse primer: 5′-GGTCCTTTTCACCAGCAAGCT-3′

The cycling conditions were 50°C for 2 min, 95°C for 10 min, and 40 cycles of 95°C for 15 s, 60°C for 1 min, and then from 60°C to 95°C for 20 min. The relative expression levels were calculated using the ΔΔCt method with the *GAPDH* and *HPRT1* genes used as internal controls. The tumours that expressed *PAX8* at a level at least 3 times higher than the HEK-293 cell (no or low expression of *PAX8*) levels were considered positive [28,29].

### Construction and transfection of siRNAs

*PAX8* siRNAs were designed following previously developed and described guidelines [30].

The sequences targeting *PAX8* were as follows:

PAX8-1: 5′-AGACAAAATTGAAGAAGAA-3′

PAX8-2: 5′-CGCCAGAACCCTACCATGT-3′

PAX8-3: 5′-TCTTTATTTATTACATGAA-3′

The other controls included:

Non-targeting 1 (NT1) to GFP: 5′-ACTACCAGCAGAACACCCC-3′

Scrambled sequence for PAX8-1 (sc8-1): 5′-AAGTTAGAAAAAAACGAAAAG-3′

Scrambled sequence for PAX8-2 (sc8-2): 5′-AACACCGGGAAACACCUTCCU-3′

All siRNAs were synthesised using the Ambion Silencer™ siRNA construction Kit (Life Technologies, Carlsbad, CA) following the manufacturer’s instructions. The control *GAPDH* siRNA template was provided with the kit. The siRNA for p53 and the non-targeting 2 (NT2) control siRNA were purchased from Qiagen (GmbH, Hilden, Germany). Two additional siRNA for p53 sc29435 (siTP53 2 in this study) and sc44218 (siTP53 3 in this study) were purchased from Santa Cruz Biotechnology (Santa Cruz, CA). Three *BCL2* siRNA were used (sc-61899) along with the control siRNA (sc-37007, NT3), and were purchased from Santa Cruz Biotechnology (Santa Cruz, CA). Two additional siRNA for BCL2 214532 (siBCL2 2 in this study) and 214533 (siBCL2 3 in this study) purchased from Life Technologies (Carlsbad, CA). All siRNAs were handled and prepared according to manufacturers’ instructions. The transfection experiments utilised A172 cells because of their endogenous *PAX8* expression, as previously described [17]. Briefly, cells were plated at densities ranging from 2 × 10^4^ to 1 × 10^5^ cells/well 24 hours prior to transfection. The siRNAs were diluted with serum-free medium to a final concentration of 10 nM and transiently transfected into cells using Lipofectamine 2000 (Life Technologies, Carlsbad, CA) or Ambion siPORT NeoFX (Life Technologies, Carlsbad, CA). The medium was replaced after 4 hours, and the cells were harvested 24–96 hours after siRNA transfection. The viable cells were counted using the trypan blue exclusion assay. Apoptotic nuclei were detected in paraffin-embedded cell clot sections using the Klenow FragEL DNA Fragmentation Kit (Merck, Darmstadt, Germany) and light microscopy. The percentage of apoptotic cells per 500 cells was measured.

### Western blot analysis

A172 protein lysates were prepared in the presence of protease inhibitors, and 100 μg protein were separated on NuPAGE 4-12% Bis-Tris Gels (Life Technologies, Carlsbad, CA). Blots were probed with primary antibodies raised against PAX8 (MRQ-50, Cell Marque), Bcl-2 (Clone 124, Dako), p53 (1C12, Cell Signaling Technology, Beverly, MA), WT1 (6FH2, Dako, Glostrup, Denmark) and β-actin (AC-15, Abcam, UK) according to the manufacturers’ instruction, or that optimised in the current study (1:200 dilution for WT1). Alkaline phosphatase conjugated antibodies were detected using the Western Breeze Immunodetection kit (Life Technologies, Carlsbad, CA).

### Statistical analysis

To analyse the PAX8-positive tumours, a comparison between the experimental groups was made using the Fisher’s exact test. For cell transfection experiments, the data are expressed as the mean ± SD, and the statistical significance was determined between the experimental groups using the Student *t* test. *P* < 0.05 was considered statistically significant and the GraphPad Prism software, version 6.00 for Macintosh (GraphPad Software, San Diego, CA) to perform all statistical tests.

## Results

### Aggressive gliomas are PAX8-positive

**Figure 1:**
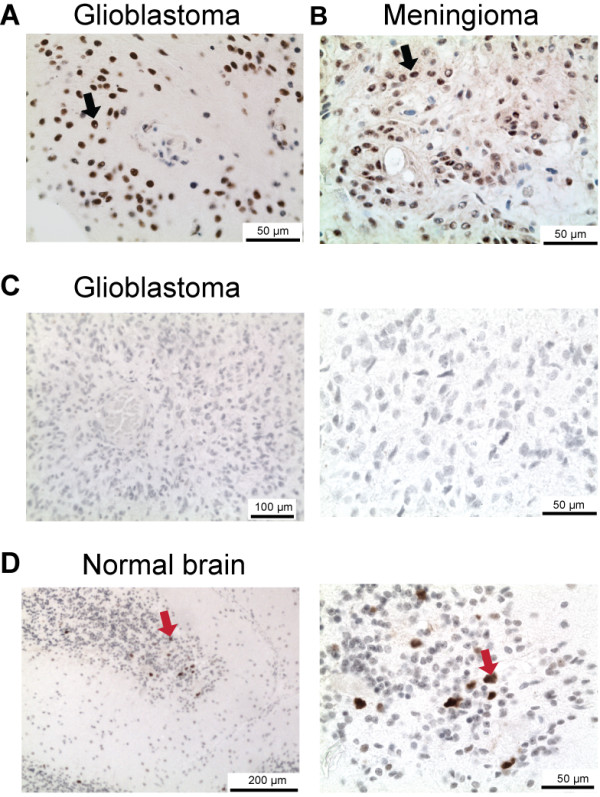
**PAX8 immunostaining in gliomas.** Representative examples of PAX8-positive and PAX8-negative immunostaining are illustrated **(A-D)**. Tissue sections were subjected to immunohistochemical stainings using an antibody raised against PAX8 (rabbit polyclonal, Cell Marque) and counterstained with hematoxylin. PAX8-positive cells were detected by light microscopy. PAX8-positive cells were detected in glioblastoma **(A)** and meningioma **(B)**. **(C)** A PAX8-negative glioblastoma was observed at low (left) and high (right) magnification. **(D)** Normal brain tissue adjacent to glioma cells was PAX8-negative except between the cerebellar molecular and nuclear layers. Examples of PAX8-positive cells are highlighted with black arrows (tumours cells) and with red arrows (non-malignant cells).

The results from the immunohistochemical analyses are presented in Table 1. PAX8-positive tumours were frequently observed in 72% (86/120) of the glioblastoma samples. The majority of the PAX8-positive glioblastomas possessed at least 60% PAX8-positive tumours cells (Figure 1A). All tumours typed as negative for PAX8 had no positive nuclear staining. PAX8-positive glioblastomas were present in all the telomere maintenance mechanism groups, but a higher prevalence was observed in the telomerase-positive (80%) and NDTMM-positive (73%) tumours compared with the ALT-positive tumours (44%; *P* = 0.007 and *P* = 0.043, respectively). All tumours had intense PAX8 immunostaining with the exception of the ALT-positive tumours, of which 4/8 ALT-positive tumours showed faint PAX8 immunostaining. Glioblastomas typed as PAX8-negative had no PAX8-positive tumour cells present (Figure 1C). PAX8-positive tumours were also observed in other aggressive tumours in the brain, including all malignant meningiomas (n = 4) and 40% of grade 3 astrocytomas (4/10).

**Table 1:** **The frequency of PAX8**- **and PAX5**-**positive brain tumours**

**Tumours type**	**n**	**PAX8-positive**	**PAX5-positive**
Glioblastoma: ALT	18	8 (44%)†	0
Glioblastoma: NDTMM	56	41 (73%)*	5 (9%)
Glioblastoma: telomerase	46	37 (80%)**	2 (5%)
Astrocytoma grade I	20	1 (5%)	0
Astrocytoma grade II	6	1 (17%)	0
Astrocytoma grade III	10	4 (40%)	0
Meningioma grade I	20	1 (5%)	0
Atypical Meningioma (Grade II)	6	0	-
Malignant Meningioma (Grade III)	4	4 (100%)	0

ALT, alternative lengthening of telomeres; NDTMM, non-defined telomere maintenance mechanism. †, four positive tumours showed faint immunostaining; *P < 0.05 and **P < 0.01 compared with ALT-positive tumours; -, not tested.

A small number of the low-grade tumours were PAX8-positive (6%, see Table 1). In the 52 cases of meningioma and astrocytomas grades I and II, three PAX8-positive cases were detected. PAX8-positive cases included one recurrent grade I astrocytoma, one grade II astrocytoma, and one meningioma (Figure 1B). All non-tumour brain cells were PAX8-negative with one notable exception. A few PAX8-positive cells were identified in the cerebellum between the molecular and the nuclear layer in the two adult brain tumours containing cerebellum tissue (Figure 1D).

All PAX8-positive tumours were verified as positive using another PAX8 antibody raised against a region of the C-terminus, which exhibits a lower homology with PAX5. An IHC analysis of PAX5 expression in the brain tumours cohort identified seven PAX5-positive tumours, all glioblastomas (Table 1).

In summary, PAX8-positive cells were detected in aggressive brain tumours. In low-grade gliomas, PAX8-positive cases were infrequently observed.

### Quantitative PCR confirms the increase of *PAX8* expression in glioblastomas

To confirm the prevalence of increased PAX8 expression in primary glioblastomas, *PAX8* expression was analyzed using QPCR in 40 glioblastoma samples (30 positive and 10 negative by IHC), recurrent pilocytic astrocytomas, PAX8-positive grade II astrocytomas, human fetal astrocytes derived from an 18-week gestated fetus (PAX8-positive control), and HEK-293 cells (no or low PAX8 expression). Fetal-derived human astrocytes had 10-fold more *PAX8* expression than the embryonic kidney cell line, HEK-293. The *PAX8* expression was 10-fold higher in 27/40 (68%) glioblastomas and the two low-grade astrocytomas typed as PAX8-positive by IHC. Three glioblastomas showed a moderate *PAX8* expression level (from 5- to 10-fold higher), three had a low *PAX8* expression level (from 3- to 5-fold higher). Seven tumours showed less than a 2-fold increase in the *PAX8* expression compared to HEK-293 cells and were typed as *PAX8*-negative tumours. In summary, all tumours typed as PAX8-positive by IHC were verified as *PAX8*-positive by QPCR.

### Glioma cell growth is inhibited by *PAX8* siRNA gene silencing

**Figure 2:**
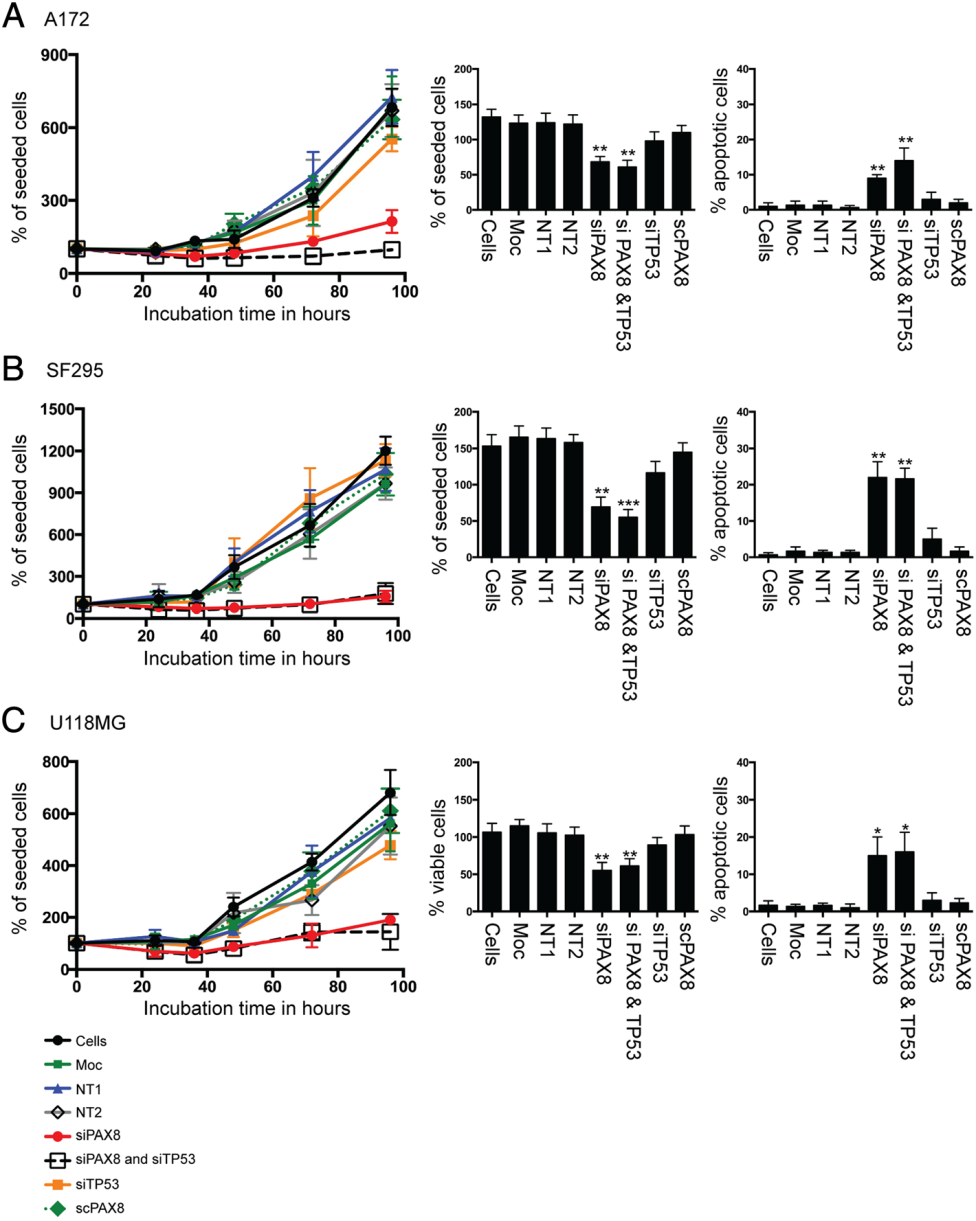
**Reduced *PAX8 *expression leads to a reduction in the glioma cell growth rate and increased glioma cell apoptosis.***PAX8* silencing reduced the cell growth rate and induced apoptosis in three glioma cell lines: **(A)** A172, with wild-type p53; **(B)** SF295, with mutant p53; and **(C)** U118MG, with mutant p53. Cells were transfected with a *PAX8* siRNA (siPAX8, PAX8-1). As controls, cells were mock-treated (Moc) or transfected with non-targeting siRNAs (NT1, NT2, NT3, and scrambled s8-1 (scPAX8)). To investigate whether the reduction in the glioma cell growth rate associated with the *PAX8*-knockdown was due to p53 function, *TP53* was also knocked down independently (sip53) or in combination with *PAX8* (siPAX8 & p53). Live cells were counted using the trypan blue exclusion assay at 24, 36, 48, 72, and 96 hours post-transfection. The percent viable and apoptotic cells 36 hours post-transfection are presented as bar graphs. *P < 0.05, **P < 0.01, and ***P < 0.001 (siPAX8 compared with scPAX8).

**Figure 3:**
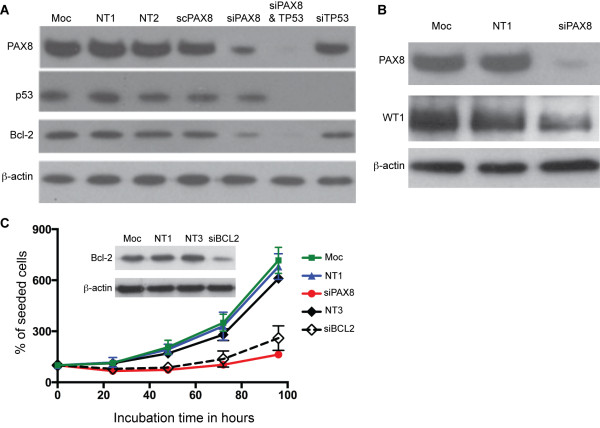
**Reduced PAX8 expression leads to decreased expression of BCL2 and WT1. (A)** The *PAX8*-knockdown (siPAX8) in the A172 glioma cell line by siRNA (PAX8-1) produced a reduction in the BCL2 expression levels. Cells lysates were prepared 36 hours after siRNA transfection, and the PAX8, BCL2, p53, and β-actin (loading control) expression levels were measured by western blot. For controls, A172 cells were transfected with mock-treated (Moc), non-targeting siRNAs (NT1, NT2, and NT3) and scrambled s8-1 siRNA (scPAX8). To ensure the reduction in the glioma cell growth rate associated with the *PAX8*-knockdown was not due to p53 function, p53 was also knocked down in A172 cells (sip53) independently or in combination with a *PAX8* siRNA (siPAX8 & p53). **(B**) The *PAX8*-knockdown (siPAX8) in the A172 glioma cell line by siRNA (PAX8-1) produced a reduction in the WT1 expression levels. **(C)** The *BCL2*-knockdown produced a similar reduction in the cell growth rate compared to *PAX8*-knockdown in the A172 glioma cell line. Cells were transfected with a *BCL2* siRNA (siBCL2) or a *PAX8* siRNA (PAX8-1, siPAX8). For controls, A172 cells were mock-transfected (Moc) or transfected with non-targeting siRNAs (NT1 and NT3). The percentage of live cells was determined by the trypan blue exclusion assay every 24 hours post-transfection. (*Insert*) Western blotting shows the BCL2-knockdown with a *BCL2* siRNA and no BCL2-knockdown in controls; the loading control is β-actin.

The silencing of *PAX8* by siRNA was performed in three glioma cell lines (A172, SF295, and U118MG) to examine whether reduced *PAX8* expression led to a reduction in glioma cell growth (Figure 2). *PAX8* was knocked-down with three distinct siRNAs (PAX8-1, PAX8-2, and PAX8-3). No significant differences in the PAX8 expression levels were detected between the different siRNA-knockdowns by western blotting (the result for PAX8-1 knockdown compared to mock-transfected, or non-targeting siRNA controls are shown in Figure 3A). The PAX8-knockdown led to a reduction in cell number in all the glioma cell lines (*P* < 0.01, *PAX8* siRNA-knockdown compared with all controls). *PAX8* silencing by siRNA produced an increase in apoptosis (Figure 2, *far right panel*) as measured by counting the apoptotic cells 36 hours post-transfection (*P* < 0.05, *PAX8* siRNA compared with all controls). To ensure the effect on cell growth was not p53 function-dependent, siRNAs to *TP53* were also transfected into the A172, SF295, and U118MG cell lines. An example of *TP53*-knockdown in A172 cells by western blotting is presented in Figure 3A. The *TP53*-knockdown was not associated with alterations in cell numbers (Figure 2). The *TP53* and *PAX8* knockdowns and cell survival studies in A172 cells were repeated using additional siRNAs (see Additional file 1).

### *PAX8* silencing leads to a reduction in tumour cell growth and reduced BCL2 expression

Because PAX8 binds to the promoter region of *BCL2* and *WT1* and enhances transcription [29,30], we investigated whether the downregulation of *PAX8* would decrease the BCL2 and WT1 expression levels in glioma cells. *PAX8* was knocked down using the PAX8-1 siRNA in A172 cells. Western blots assessing the relative levels of BCL2 with PAX8 knockdown revealed a reduction in the BCL2 expression (Figure 3A), whereas in the controls (mock transfection, transfection with non-targeting siRNAs or the scrambled siRNA [sc8-1]) no reduction in PAX8 or BCL2 expression was observed (Figure 3A). A similar result was found for WT1, in which reduced WT1 was specific to lysates with PAX8 knockdown (Figure 3B). These data suggest that *PAX8* silencing leads to downregulation of BCL2 and WT1 expression.

To investigate whether this reduction in BCL2 expression could explain the growth reduction associated with the *PAX8*-knockdown, BCL2 was knocked down using a *BCL2* siRNA in A172 cells, and cell growth monitored for 24–96 hours after transfection. *BCL2* silencing resulted in a reduction in glioma cell growth similar to the reduction observed with *PAX8* silencing (Figure 3C) at 48–96 hours post-transfection (*P* < 0.01, *BCL2* siRNA compared with controls). This observation provides further evidence that the effect of PAX8 on *BCL2* expression is responsible for the alterations in glioblastoma cell growth [31,32]. The *BCL2* knockdown and cell survival studies in A172 cells was repeated using additional siRNAs (see Additional file 1).

## Discussion

The current study represents the first extensive analysis of the PAX8 expression levels in gliomas. Our data showed that PAX8 is increased in most high-grade gliomas and is a pro-survival factor for glioma cells. In another study with a large tumour panel, PAX8-positive tumours were frequently detected in renal cell carcinomas (90%), thyroid cancers (90%), endometrial cancers (84%), cervical adenocarcinomas (83%), and ovarian cancers (79%) [33]. Our data include glioblastoma and malignant meningioma amongst the cancers with a high incidence of PAX8-positive tumours. PAX8 transactivates the promoters of the telomerase catalytic subunit (hTERT) and the telomerase RNA component (hTR) to increase telomerase activity [17], and as might be predicted, the majority of the telomerase-positive tumours were also PAX8-positive. Therefore, in telomerase-positive glioblastomas, the *PAX8* expression may play an important part in the immortalisation process by regulating telomerase activity. But *PAX8* expression was not restricted to telomerase-positive glioblastomas. The frequency of PAX8-positive tumours was similar between the telomerase- and NDTMM-positive tumours and was lower in the ALT-positive glioblastomas (44% of tumours, of which only half showed strong PAX8 immunostaining).

In cancer, the over-expression of the *PAX* genes is often attributed to chromosomal rearrangements that result in fusion proteins [7,10,34,35]. In thyroid adenocarcinomas the PAX8/PPAR-γ (peroxisome proliferator-activator receptor gamma 1) fusion protein confers many oncogenic properties, including increased proliferation, decreased apoptosis and the inhibition of wild-type PPAR-γ [7,36-38]. The cause for the increased *PAX8* expression in glioblastomas is unknown. In gliomas, the chromosome 2q13 locus, where the *PAX8* gene is located, is not a glioma susceptibility locus (OMIM #137800), but other mechanisms for the increased *PAX* expression in cancer have been described. Hypomethylation, for example, produces an increase in *PAX2* expression in endometrioid carcinoma [9]. In adult tissues, the *PAX* genes are proposed to be important for maintaining stem cells; therefore, the increased PAX8 expression in glioblastomas may be indicative of an early cell lineage [39]. Additionally, co-activators of PAX8 are increased in gliomas. Increased TAZ (transcriptional co-activator with PDZ-binding motif) is observed in the mesenchymal subtype of glioblastoma [40,41]. Furthermore, TAZ is reduced in proneural glioblastomas, which are usually ALT-positive tumours and those with reduced PAX8 positivity in the current study [41].

In low-grade gliomas, PAX8 was not detected in the majority of tumours. A reduction of the PAX8 expression levels in low-grade tumours is consistent with the association of PAX8 expression with more aggressive tumours. Our results are also consistent with another study in which the transcriptional target of PAX8, *WT1*, was decreased in low-grade compared with high-grade gliomas [20]. A larger cohort of the low-grade PAX8-positive tumours might show an association with poorer outcomes because in our cohort, the single PAX8-positive grade I astrocytoma was a recurrent tumour.

In non-malignant cells, PAX8 expression was detected in a minimal number of cerebellar cells. Otherwise, all other non-malignant cells examined were PAX8-negative. The virtual absence of *PAX8* in the adult brain is consistent with studies in mice in which the adult murine brain expressed *PAX8* at levels no higher than the background signal [16]. PAX8 expression in the adult human brain has not been previously studied, and our results suggest that the residual PAX8 expression does occur in a small minority of cells. The predominance of high-grade gliomas expressing high levels of PAX8 and the virtual absence of PAX8 expression in normal brain makes PAX8 signalling an appealing therapeutic target pathway.

We found that PAX8 acted as a pro-survival factor for glioblastomas. The silencing *PAX8* in several glioma cell lines caused a marked reduction in cell number, which is partly explained by an increase in apoptosis. Reduced PAX8 expression produced a reduction in the BCL2 expression levels, and *BCL2* inhibition by siRNA-knockdown reduced the glioma cell growth rate. These findings are consistent with previous reports that demonstrate *PAX* expression enhances cell growth and survival [15,42], and upregulated BCL2 is found in gliomagenesis [21,31]. In other studies *BCL2* silencing induced cell death *in vitro*[43,44], led to an arrest of cell cycle progression [19], and was associated with the downregulation of multiple developmental genes [45].

## Conclusions

Increased PAX8 expression was frequently detected in high-grade gliomas. This finding combined with the reduction in glioma cell growth caused by a reduction in Bcl-2 expression after *PAX8*-knockdown and the small amount of PAX8 expression in normal brain tissue suggests that *PAX8* would be a suitable target pathway for glioma therapy.

## Competing interests

The authors have no competing interests.

## Authors’ contributions

NH, Histo-pathological assessment, interpretation of the results and writing of the manuscript; YJC, quantitative PCR and knockdown experiments, and interpretation of the results; AT, MO, RB, BB, and MM the collection and selection of tumours, patient consenting, and collecting and interpreting clinical data, interpretation of the results and writing of the manuscript; TW, quantitative PCR and interpretation of the results; AW, AS, and RE telomere maintenance mechanism typing, immunohistochemistry staining, and interpretation of slides; ME and AB, *in vitro* experimental design and interpretation of results; JR, project conception, experimental design, interpretation of the results and writing of the manuscript; TS, Histo-pathological assessment, knockdown experiments, project conception, experimental design, interpretation of the results and writing of the manuscript. All authors read and approved the final manuscript.

## Pre-publication history

The pre-publication history for this paper can be accessed here:

http://www.biomedcentral.com/1471-2407/14/159/prepub

## Supplementary Material

Additional file 1 **Validation of cell survival results using additional siRNAs to PAX8, TP53, and BCL2. (A)** Additional siRNAs (PAX8 2–3, TP53 2–3, and BCL2 2–3) in the A172 glioma cell line by siRNA produced a reduction in the corresponding protein level. Cells lysates were prepared 36 hours after siRNA transfection, and the PAX8, BCL2, p53, and β-actin (loading control) expression levels were measured by western blot. For controls, A172 cells were transfected with mock-treated (Moc), non-targeting siRNAs (NT1, and NT3). **(B)** To ensure the additional siRNAs had the same affect on cell survival the A172 cell growth rate was measured for *PAX8*-knockdown with siRNA 2 and 3 (top panel), TP53 siRNA 2 and 3 (top panel), and BCL2 siRNA 2 and 3 (bottom panel). The results were similar to that found with the original si RNAs (PAX8-1, TP53-1, and BCL2-1 Figures 2 and 3). For controls, A172 cells were mock-transfected (Moc) or transfected with non-targeting siRNAs (NT1 for PAX8 and TP53 knockdowns and NT3 for BCL2 knockdowns). The percentage of live cells was determined by the trypan blue exclusion assay every 24 hours post-transfection. Click here for file
