# Supplementary figures and images for: Increased paired box transcription factor 8 has a survival function in Glioma
Source: BMC Cancer. 2014 Mar 6;14:159. doi: 10.1186/1471-2407-14-159 (PMC4015841; doi:10.1186/1471-2407-14-159)

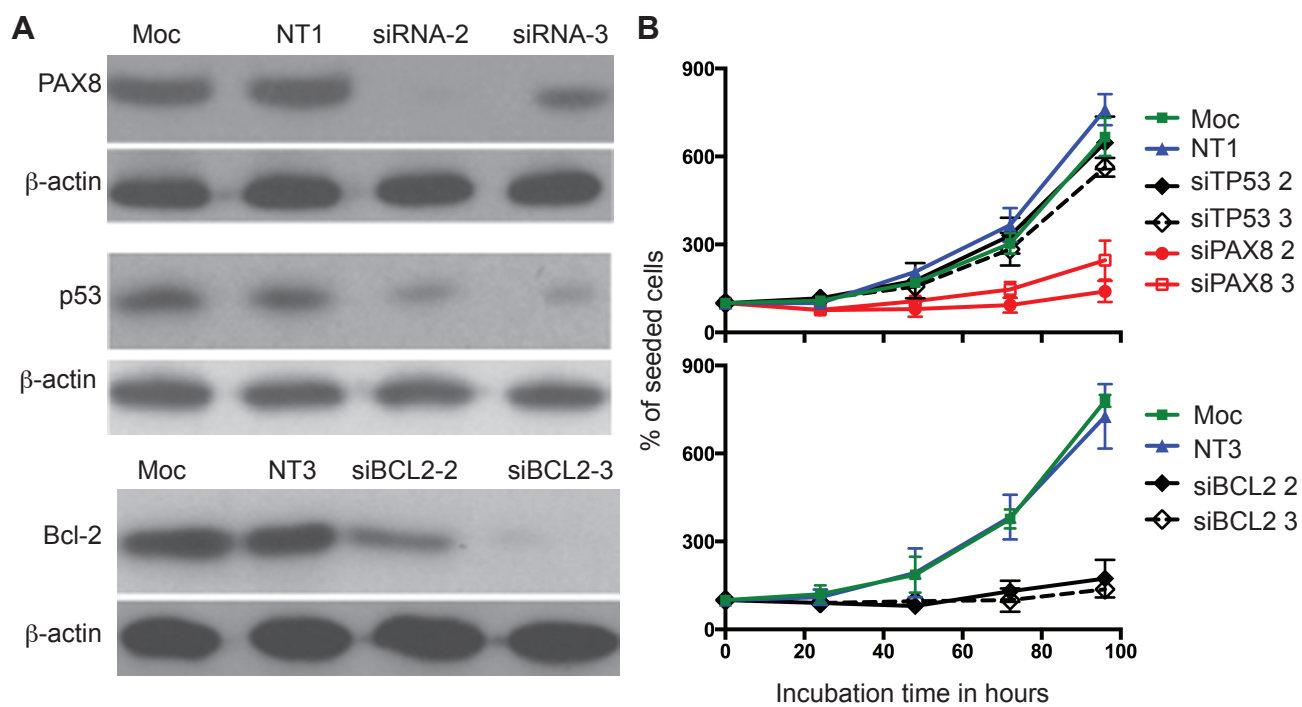

*Additional file 1*

Supplement: Additional file 1 — Validation of cell survival results using additional siRNAs to PAX8, TP53, and BCL2. (A) Additional siRNAs (PAX8 2–3, TP53 2–3, and BCL2 2–3) in the A172 glioma cell line by siRNA produced a reduction in the corresponding protein level. Cells lysates were prepared 36 hours after siRNA transfection, and the PAX8, BCL2, p53, and β-actin (loading control) expression levels were measured by western blot. For controls, A172 cells were transfected with mock-treated (Moc), non-targeting siRNAs (NT1, and NT3). (B) To ensure the additional siRNAs had the same affect on cell survival the A172 cell growth rate was measured for PAX8-knockdown with siRNA 2 and 3 (top panel), TP53 siRNA 2 and 3 (top panel), and BCL2 siRNA 2 and 3 (bottom panel). The results were similar to that found with the original si RNAs (PAX8-1, TP53-1, and BCL2-1 Figures 2 and 3). For controls, A172 cells were mock-transfected (Moc) or transfected with non-targeting siRNAs (NT1 for PAX8 and TP53 knockdowns and NT3 for BCL2 knockdowns). The percentage of live cells was determined by the trypan blue exclusion assay every 24 hours post-transfection. [file 1471-2407-14-159-S1.pdf]
